# MECHANISMS OF RADIATION CARCINOGENESIS: WHAT IS REALLY INDUCED?

**DOI:** 10.1093/rpd/ncac063

**Published:** 2022-09-09

**Authors:** Nori Nakamura

**Affiliations:** Radiation Effects Research Foundation, Department of Molecular Biosciences, 5-2 Hijiyama Park, Minami-ku, Hiroshima 732-0815, Japan

## Abstract

It has been difficult to understand why the relative risk for cancer decreases with an increase in time since an exposure to radiation. It was recently recognized that this decline can be explained by a parallel shift of the age-related cancer mortality curve toward younger ages. In fact, it has been known for many years that mouse survival curves exhibit a parallel shift toward younger ages following an exposure to radiation, but it was not recognized that the mutation induction theory is incompatible with this parallel shift. This is because a parallel shift in the survival curve implies that all the irradiated individuals are affected, but the mutation induction theory assumes that only a fraction of the irradiated individuals is affected following an exposure to radiation. Thus, it seems likely that the target of radiation action, which leads to carcinogenesis, is not restricted to epithelial cells but includes all of the surrounding cells leading to an altered microenvironment. Since it is repeatedly observed that radiation-exposed normal tissues can stimulate transplanted or spontaneously arising tumor cells to grow faster, worsen the malignant phenotypes and finally kill the host earlier than usual, an exposure to radiation seems most likely to cause tissue inflammation, which creates conditions favorable for the growth of spontaneously arising tumor cells. This new concept suggests that it might be possible to attenuate the extent of radiation carcinogenesis by intervening in tissue inflammatory processes.

## INTRODUCTION

First, it should be mentioned that this review paper was presented at the International Symposium; Environmental Dynamics of Radionuclides and Biological Effects of Low Dose-Rate Radiation, which was held in Aomori, Japan in 27–29 September 2021, organized by the Institute for Environmental Sciences. The content is largely composed of two previous review articles^(1, 2)^.

**Figure 1:**
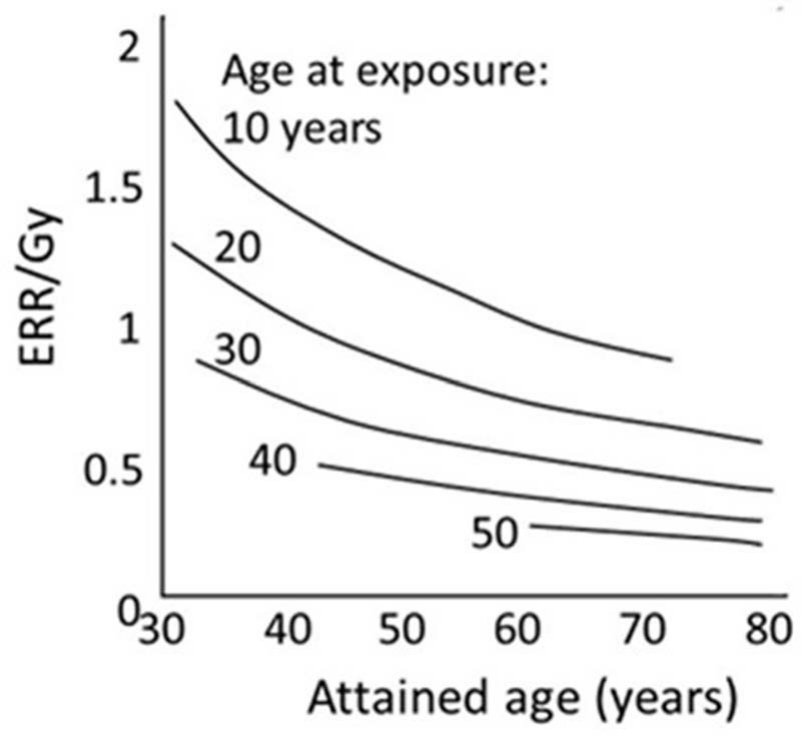
Time course of excess relative risk (ERR) per Gy in relation to an increased number of years since an exposure to A-bomb radiation. Each curve represents a group who was exposed at different ages from 10 to 50 y^(3)^.

It is well known that an exposure to radiation is hazardous because it may increase the risk of developing cancer. However, not much is known about how an exposure to radiation results in this increased risk. It has been difficult to understand the epidemiologic data from atomic-bomb (A-bomb) survivors, which indicates that the relative risk (RR) decreases with an increase in years since an exposure (Figure 1). To date, there has been no explanation given for this decline, which leads to the question: is this declining trend *by chance* or *a natural consequence* of radiation exposure?

**Figure 2:**
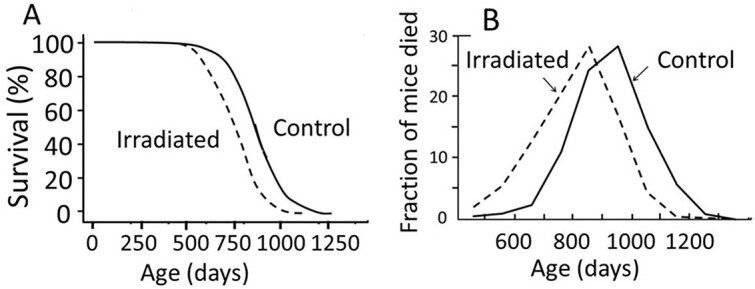
(**A**) Survival curves of control and irradiated mice^(4)^. (**B**) Fraction of deceased animals resulting from all neoplasms in every 100-d window^(5)^ (reproduced from ref. 1 with permission).

**Figure 3:**
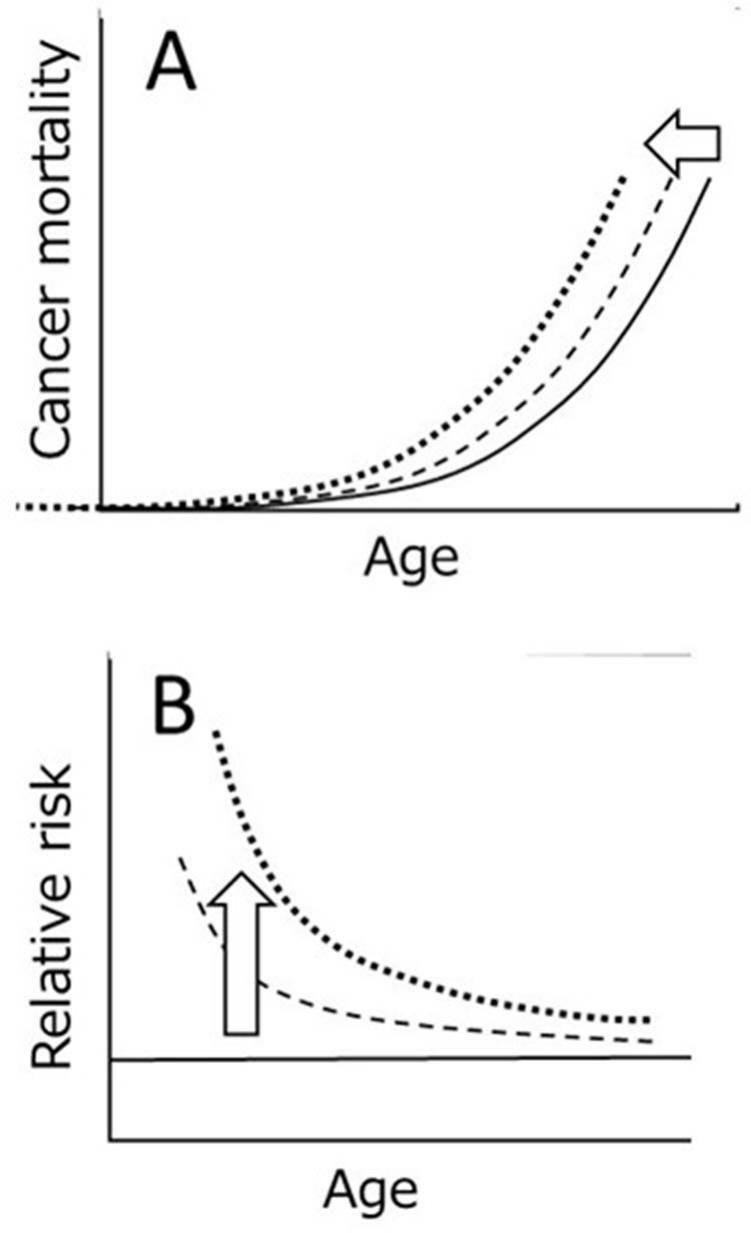
An artificial shifts of a cancer mortality curve toward younger ages (panel A) result in temporal changes of RR values with higher values at younger ages followed by a continuous decline with an increase in age (panel B).

**Figure 4:**
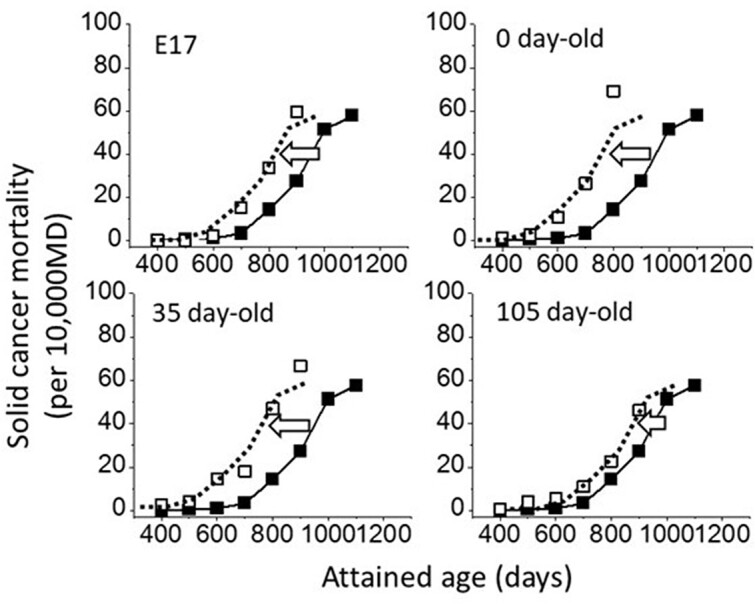
Solid tumor mortality of mice irradiated to 1.9 Gy of gamma rays at different ages from E17, at birth, and at 35 and 105 d^(6)^. E17 stands for embryonic Day 17. Closed symbols represent the control group and the open symbols represent the irradiated group.

## A SHIFT IN THE CANCER MORTALITY CURVE CAN EXPLAIN A DECLINE IN THE RR

It can be seen that the survival curves of irradiated mice can shift toward younger ages in parallel with the control curve (Figure 2A). This means that all the irradiated animals were equally affected because the distribution of individual deviations from the mean life span remains the same following an exposure to radiation while the mean lifespan is reduced (Figure 2B). However, this notion contradicts the theory of mutation induction in radiation carcinogenesis because this theory assumes that a fraction of the irradiated animals is affected, and that the fraction increases with an increase in the radiation dose.

Following the suggestion that the irradiated animals die earlier than control animals, the baseline cancer mortality curve (e.g. *y* = *ax*^5^) was shifted toward younger ages to see the behavior of RR by taking the ratios of the mortality between the shifted and non-shifted curves (Figure 3). The results showed that the RR simply declines with an increase in the number of years since an exposure to radiation. (The early phase with a sharp decline of RR is usually unseen because it is still within the cancer latency period.) Now, the declining trend of RR can make sense because the elevated RR and its declining trend are most likely caused by an earlier shift of the mortality curve. In other words, the declining trend of RR is not caused by chance but is *a natural consequence* of radiation exposure.

Figure 4 shows the results reported by Sasaki and Fukuda in 2005, a unique set of data, which showed solid tumor mortality in mice irradiated at various ages^(6)^. Unfortunately, the authors presented the mortality data in several tables but not in any graphs. Here, some of the data are plotted in figures: The closed symbols represent control group and the open symbols are the data from irradiated animals. The dotted lines represent control curves shifted toward younger ages and fitted by eye. It is striking to see how well the shifted curves closely fit the data. In contrast, it can easily be seen that explaining the data for the irradiated group is difficult if the control curve is shifted upwards in a manner, which depends on both the attained age and age at exposure (i.e. by the mutation induction theory).

## THE EARLIER ONSET MODEL FITS A-BOMB SURVIVOR DATA

**Figure 5:**
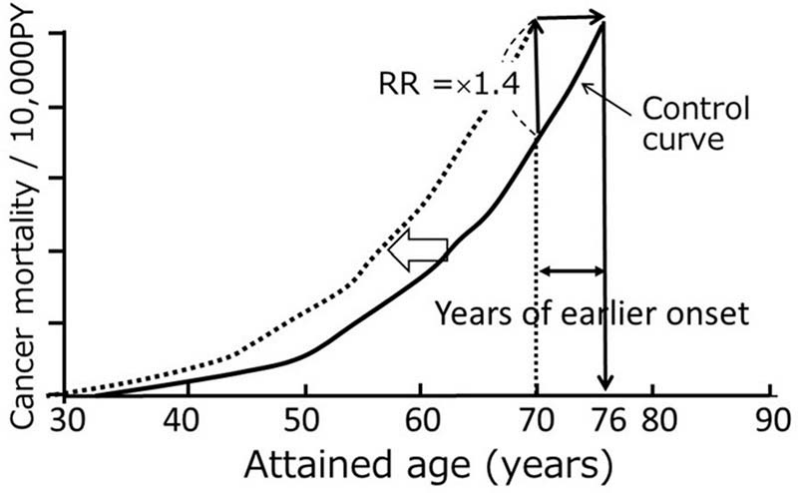
A model to estimate the number of years of an earlier onset of cancer. Epidemiologically estimated RR value per Gy for those who were exposed to the bomb at age 30 and reached age 70 is 1.4, which was used to estimate the age of the control group at which this 1.4-fold increase would be attained (reproduced from ref. 1 with permission).

**Figure 6:**
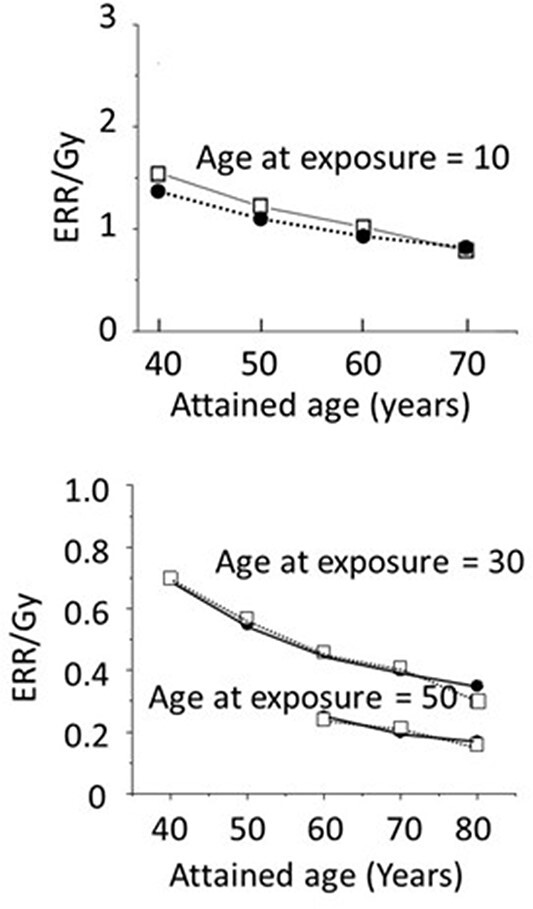
Simulated and epidemiologically estimated ERR/Gy for those who were 10, 30 and 50 y old at the time of an exposure. Open symbols indicate simulated results, and the closed symbols indicate epidemiologically estimated ERR (reproduced from ref. 1 with permission).

Because the mouse data appeared to suggest that an exposure to radiation causes an earlier onset of malignancies, an attempt was made to see if this model might explain A-bomb survivor data. Figure 5 shows the approach used. For example, for those who were exposed at age 30, it was reported that the RR is 1.4 when they reached age 70. Thus, the solid cancer mortality of the control group at age 70 was first calculated, which was then multiplied by 1.4. Subsequently, it was calculated the age of control subjects at which this 1.4-fold increased mortality is attained. This was 76 y of age, which is interpreted to indicate that those who were exposed to 1 Gy at age 30 could have experienced an earlier death from cancer by ~6 y. And, the ratio of the mortality between the shifted and control curves represents the RR at different attained ages. Figure 6 represents the results of thus calculated RR along with the epidemiologically estimated RR.

In Figure 6, the *Y* axis indicates excess RR (ERR) per Gy, and the *X* axis represents attained age. Open symbols indicate simulated results acquired with the approach shown in Figure 5, and the closed symbol indicate epidemiologically estimated ERR. The sex-averaged mean of years of earlier death per 1 Gy is ~10, 6 and 3 y for those who were 10, 30 and 50 y old at the time of the bomb, respectively. It is impressive to see that an appropriate shifting of the control mortality curve toward younger ages could faithfully reproduce the temporal changes of RR.

## THE TARGET OF A RADIATION EXPOSURE IS UNLIKELY TO BE EPITHELIAL CELLS

**Figure 7:**
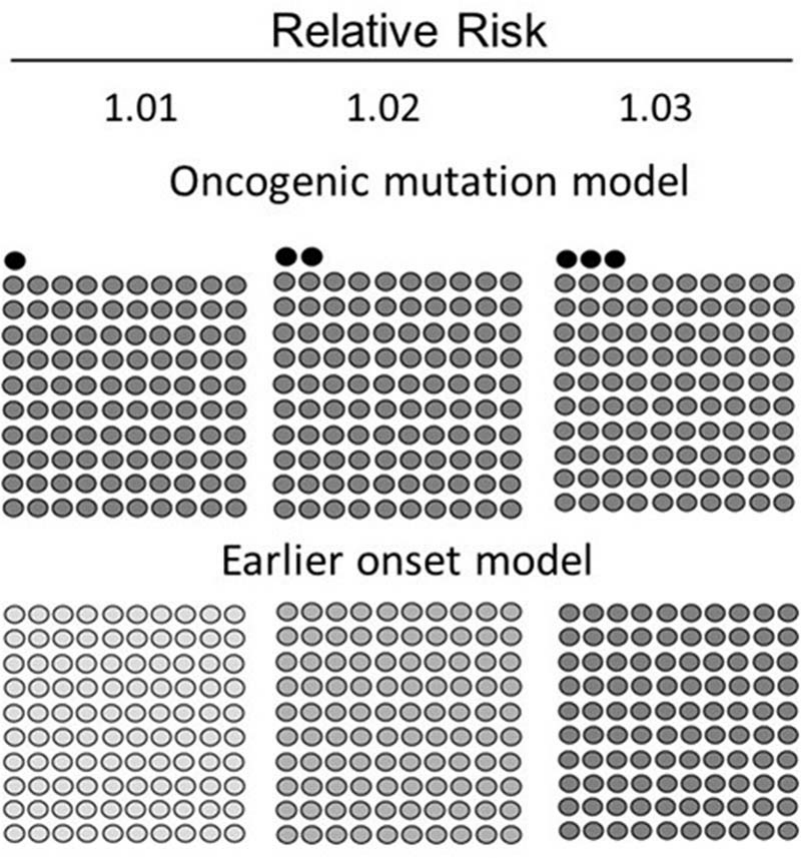
A schema to indicate a paradigm shift in thinking about the mechanism of radiation carcinogenesis (reproduced from ref. 1 with permission).

Figure 7 shows a schema, which indicates why it is necessary to move away from mutation induction theory of radiation carcinogenesis. The mutation induction theory assumes that a fraction of the irradiated individuals is affected. As shown in the upper row panels (oncogenic mutation model), when the RR increases from 1.01 to 1.03, it is assumed that one to three excess cancers (black circles) would be present when 100 spontaneous cancers (gray circles) occur. However, this model is incompatible with the parallel shift of an entire mouse survival curve. Instead, we need to think of the model shown in the lower row panels (earlier onset model) where the increased shading of all cancers with the increasing RR indicates that all cancers in the population are affected and that the dose effect is expressed as an increase in the degree of life shortening (i.e. time matters) and not as an increased number of the affected individuals. In such a situation, the target of a radiation exposure cannot be restricted to epithelial cells alone but includes all the surrounding cells.

**Figure 8:**
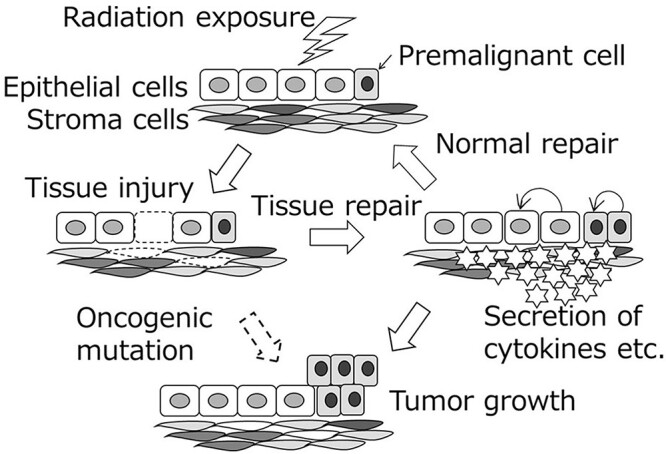
A model for radiation-induced increased cancer risk, which is derived from tissue damage and its repair. The inflammatory microenvironment encourages transformed cells to form a tumor (reproduced from ref. 1 with permission).

Figure 8 shows a model of radiation-induced tissue damage and subsequent secretion of various inflammatory substances, which help not only to repair the damaged tissue, but also support spontaneously arising malignant cells to form a tumor earlier than if they were not exposed to radiation.

## PATHS TOWARD TISSUE INFLAMMATION

**Figure 9:**
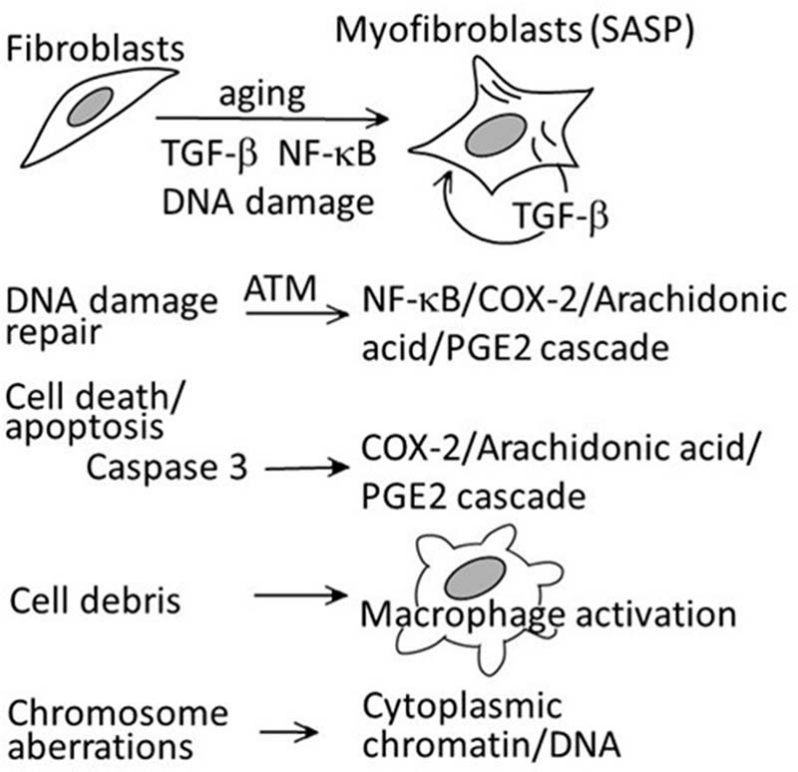
Multiple pathways, which may lead to tissue inflammation. See reference 2 for more details.

There are at least five major pathways, which may lead to the creation of a microenvironment, which supports tumor cell growth (Figure 9). For further details, see reference 2.

- The first pathway is to activate fibroblasts or stroma cells. TGF-β, NF-κB and unrepaired DNA damage are involved here.
- The second pathway is related to DNA damage and repair, which involves the activation of ATM. This activation of ATM leads to activation of NF-κB and to increased levels of downstream COX-2, and finally of prostaglandin E2 (PGE2).
- The third pathway is related to apoptotic cell death, in which caspase 3 conducts the job, and activation of caspase 3 leads to COX-2 activation followed by the same pathway mentioned above.
- The fourth pathway is related to macrophage activation since cellular debris needs to be somehow cleared *in vivo*, and activated macrophages release various inflammatory factors to attract immune cells to the site.
- The fifth pathway is related to DNA or chromosome breaks. If cells bearing broken or damaged chromosomes or DNA have replicated, chromatin fragment that are not coated with nuclear membrane may be located in the cytoplasm, and this would be indicative of a viral infection in the cells and result in the recruitment of immune cells to eradicate the damaged cells.

## RADIATION EFFECTS IN NORMAL TISSUES

There are many publications, which indicate that irradiated normal tissues can create a microenvironment, which is favorable for tumor cell growth and malignant transformation.

**Figure 10:**
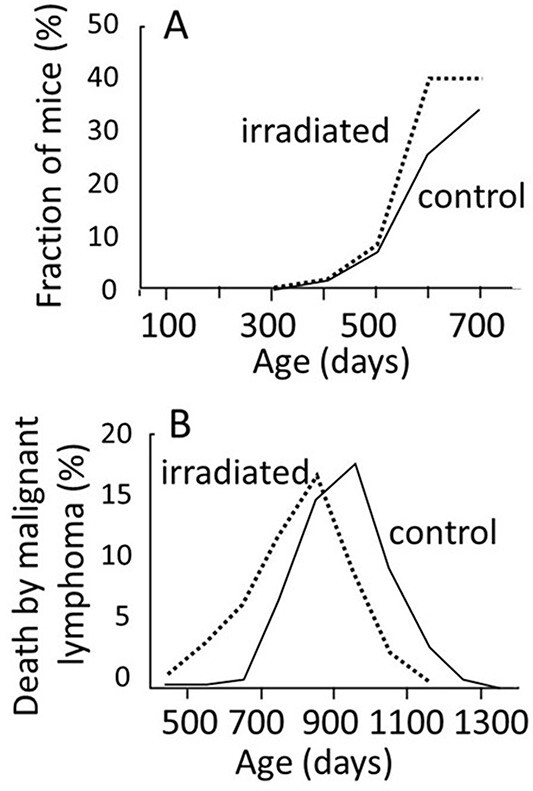
(**A**) The fraction of mice bearing malignant lymphoma (findings from sacrificed animals)^(7)^. (**B**) The fraction of animals died from malignant lymphoma^(5)^. Although there seems no earlier occurrence of the lymphoma, death from the disease occurred earlier by ~100 d in the irradiated group.

- The first example is related to malignant lymphoma. Figure 10 represents data from this disease in mice from Tanaka *et al*.^(5, 7)^. Panel A shows the incidence of malignant lymphoma when mice were serially sacrifice every 100 d^(7)^. Solid line indicates the control group, and the dotted line indicates the irradiated group (animals were exposed to a low-dose-rate exposure for 400 d to reach a total dose of 8 Gy). Because this is not an autopsy, the examined animals were not deceased but sacrificed, and the results indicate that an exposure to radiation did not lead to an earlier appearance of malignant cells. In contrast, panel B shows the results of autopsies (a pathology examined after death), and the exposure clearly caused earlier deaths from malignant lymphoma by ~100 d^(5)^. The two panels indicate that tumor cells start to appear at approximately the same age in both irradiated and control animals, but tumor growth is faster in the irradiated animals and more likely to kill the host than in non-irradiated animals.
- The second example is thymic lymphoma in the mouse. Under a specific irradiation protocol using a lymphoma-prone strain of mice, thymic lymphoma develops. However, if the thymus was surgically removed prior to irradiation, thymic lymphoma does not occur. Then, if a newborn thymus is transplanted into the thymectomized mice, thymic lymphoma develops but most of them are derived from the transplanted cells from the non-irradiated donors^(8, 9)^.
- The third example is that irradiation of the hind legs of mice with doses up to 5 Gy can help transplanted tumor cells to grow faster. If the transplantation is delayed by 3 d, the growth supporting effect is greater, which indicates that recovery from radiation-induced tissue injuries progress slowly^(10)^.
- Another example is that pre-leukemic cells can be transformed into malignant form following transplantation into irradiated mice^(11)^.
- Further, an exposure to radiation activates TGF-β and NF-κB, which leads to activation of downstream IL-33. And both TGF-β and IL-33 promote regulatory T cells (T-reg cells), which suppress cytotoxic CD8^+^ cells and create an immunosuppressive microenvironment, which would be favorable for the transformed cells to persist and expand^(12, 13)^.

## NEW EXPLANATIONS FOR OLD OBSERVATIONS

Past unexplained biologic effect of radiation may now be explained from a new standpoint. For example, the dose-rate effect in radiation carcinogenesis could be a matter of balance between induction of inflammation and its healing. The age-at-exposure effect (i.e. young individuals are more sensitive than old ones) can now be understood, at least partly, because young tissues can produce larger quantities of inflammatory factors^(14)^. At low doses, there will be no excess cancers, but rather a loss of days or weeks of life if an individual dies from cancer.

Specifically, as mentioned in Figure 6 and its explanation, an exposure to 1 Gy of acute gamma rays caused earlier deaths from cancer by ~10, 6 and 3 y for those who were 10, 30 and 50 y old at the time of the exposure, respectively. Because mouse data have repeatedly shown that the life-shortening effect of radiation is linearly related to the dose, one may calculate the life-shortening effect of radiation of acute 20 mGy as 1/50 of the effect, which is caused by 1 Gy; namely 3–10 weeks of life lost (Table 1) under the assumption of a linear non-threshold response.

**Table 1:** Comparisons of cancer risks

Age ATB*	RR at age 70		Loss of life	
	1 Gy	20 mGy	1 Gy	20 mGy
10	1.81	1.016	10 y	10 weeks
30	1.42	1.008	6 y	6 weeks
50	1.21	1.004	3 y	3 weeks

^*^ATB: at the time of the bomb.

## RADIATION CARCINOGENESIS IS LIKELY TO BE PREVENTABLE

**Figure 11:**
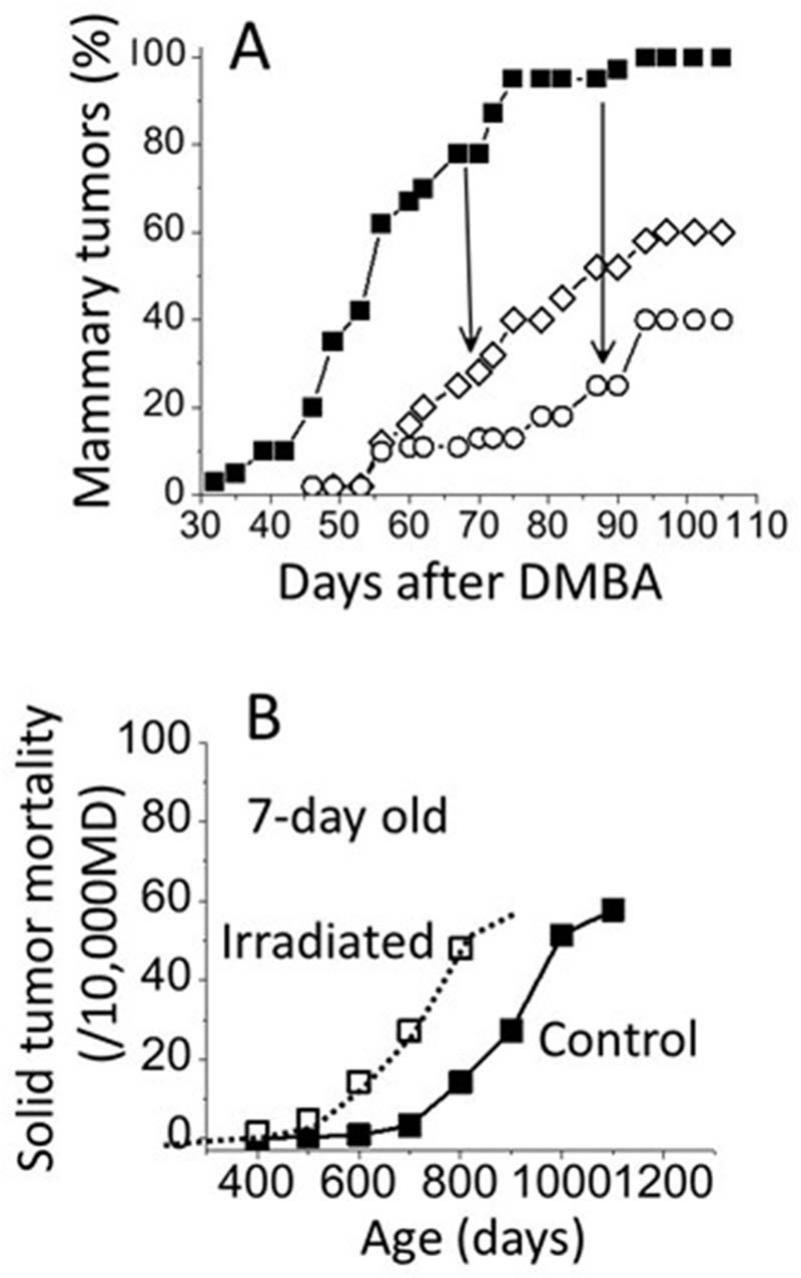
**(A**) Induction of mammary tumors in rats treated with DMBA (black square)^(15)^. Open symbols indicate animals fed with ibuprofen (white diamond; an inhibitor of COX-1 and -2) or celecoxib (white circle; an inhibitor of COX-2) following the administration of DMBA. (**B**) Solid tumor mortality of mice irradiated at Day 7 with 1.9 Gy of gamma rays^(6)^.

Figure 11 shows that radiation is not a potent carcinogen when compared with other potent chemical carcinogens, for example, 7,12-dimethylbenz-[α]anthracene (DMBA).

DMBA-induced mammary tumors start to appear 30 d following its administration, and the frequency reaches 100% in as soon as within 100 d (Figure 11A)^(15)^. In contrast, 1.9 Gy of radiation delivered to 7-d-old mice, the most sensitive stage for life shortening by tumors, may show an increase in the risk only after 400 d following an exposure (Figure 11B)^(6)^. In addition, it is quite noticeable that the carcinogenic effect of DMBA is attenuated substantially by feeding the animals with a diet, which contains ibuprofen (an inhibitor of both COX-1 and COX-2) or celecoxib (an inhibitor of COX-2) to reduce the production of PGE2. These observations strongly indicate that it is likely that the carcinogenic effects of radiation, which are far weaker than that of DMBA, could also be inhibited or blocked. It is hoped that blocking or preventing the carcinogenic effects of radiation will become an active and exciting area of research in radiation biology in the 21st century.

## EXCEPTIONS

It should be noted that the present hypothesis is based on the fact that cancer mortality increases exponentially in most tissues with an increase in age. However, there are childhood cancers and some types of leukemia that arise quite early following an exposure to radiation. These tumors are more likely to be caused by the induction of mutations and are not considered in the model presented here.
